# A hybrid unsupervised machine learning model with spectral clustering and semi-supervised support vector machine for credit risk assessment

**DOI:** 10.1371/journal.pone.0316557

**Published:** 2025-01-10

**Authors:** Tao Yu, Wei Huang, Xin Tang, Duosi Zheng

**Affiliations:** 1 School of Mathematics, Harbin Institute of Technology, Harbin, China; 2 Department of Mathematics, Southern University of Science and Technology, Shenzhen, China; 3 College of Business, Southern University of Science and Technology, Shenzhen, China; 4 National Center for Applied Mathematics Shenzhen, Southern University of Science and Technology, Shenzhen, China; University of Roehampton - Digby Stuart College, UNITED KINGDOM OF GREAT BRITAIN AND NORTHERN IRELAND

## Abstract

In credit risk assessment, unsupervised classification techniques can be introduced to reduce human resource expenses and expedite decision-making. Despite the efficacy of unsupervised learning methods in handling unlabeled datasets, their performance remains limited owing to challenges such as imbalanced data, local optima, and parameter adjustment complexities. Thus, this paper introduces a novel hybrid unsupervised classification method, named the two-stage hybrid system with spectral clustering and semi-supervised support vector machine (TSC-SVM), which effectively addresses the unsupervised imbalance problem in credit risk assessment by targeting global optimal solutions. Furthermore, a multi-view combined unsupervised method is designed to thoroughly mine data and enhance the robustness of label predictions. This method mitigates discrepancies in prediction outcomes from three distinct perspectives. The effectiveness, efficiency, and robustness of the proposed TSC-SVM model are demonstrated through various real-world applications. The proposed algorithm is anticipated to expand the customer base for financial institutions while reducing economic losses.

## 1 Introduction

In the era of big data, developments in artificial intelligence such as machine learning offer several benefits to many different sectors [1]. Machine learning research has increasingly focused on credit classification as a primary method for assessing credit risk. To avoid potential default risk and pursue considerable profit, banks and lending institutions are encouraged to accurately screen out borrowers with strong repayment capabilities when approving loans. Financial institutions use credit classification methods to discern between “good” and “bad” loan applicants [2]. This differentiation is vital as good customers are more likely to repay their loans promptly, benefiting financial institutions [3]. Conversely, bad customers pose a risk of financial loss. Therefore, the development of efficient and accurate credit classification models is essential, as even marginal enhancements can significantly impact financial outcomes [4]. Recent advancements in machine learning and data mining have streamlined financial decision-making, reducing credit analysis costs and expediting loan approvals [5]. Various machine learning methodologies, such as support vector machines (SVM), decision trees (DT), and K-nearest neighbors (KNN), have proven effective in credit classification [6–9].

The traditional classification problem pertains to supervised binary classification. However, the process of labeling data points is time-consuming and labor-intensive, especially given the large amount of unlabeled data in real-life settings [10, 11]. Financial institutions and banks can significantly enhance their business scope and improve prediction accuracy by effectively leveraging these unlabeled credit data. The unsupervised algorithm can analyze unlabeled samples when a new institution receives a credit application from a lender, thus reducing the financial institution’s time and data acquisition costs. Unsupervised classification algorithms, also known as clustering methods, are designed to solve classification tasks in unlabeled datasets [10, 12]. Unsupervised learning have been investigated in credit card fraud detection and money laundering detection [13]. On the other hand, unsupervised classification algorithms can help improve supervised learning accuracy [13–15]. The primary objective of unsupervised classification is to uncover patterns and relationships within the data. Various advanced unsupervised classification algorithms have been established, including K-means, self-organizing maps, spectral clustering, and SVM-based unsupervised methods [16–19]. Despite their efficiency, the accuracy of these unsupervised classification algorithms is limited when applied to imbalanced datasets, where the number of samples in different classes differ considerably in the context of binary classification tasks [20].

Notably, dataset imbalance may cause unsupervised classification algorithms, such as K-means and KNN, to converge to local optimal, and undermine their robustness [21]. In credit risk assessment, the number of non-defaulting borrowers typically exceeds that of defaulting borrowers [5]. Consequently, credit risk datasets are typically imbalanced, with a clear dominance of the majority class over the minority class. For example, when financial institutions develop new businesses, there may be a lack of relevant labeled data to train classification models. In such cases, credit assessments on loan applications are performed using unsupervised classification algorithms. However, there may typically only be 20 defaulters among 100 loan applicants. Traditional unsupervised classification has been noted to be inefficient in identifying minority classes in such imbalanced datasets [22], potentially resulting in significant financial losses for institutions relying on these models. Because the imbalanced ratio of the datasets will negatively affect the prediction accuracy of traditional unsupervised algorithms for minority class samples. The existing research has not comprehensively addressed the imbalance problem in unsupervised classification, given the following two challenges. First, analyzing distribution differences resulting from imbalances is difficult without labeled data [23]. As a result, clustering algorithms create clusters of similar size. It is common for large and small groups to make observations at the same time. Second, compared with supervised algorithms, unsupervised algorithms are less prone to overfitting when applied to imbalanced datasets but still cannot efficiently recognize minority-class samples. In other words, some unsupervised algorithms can fall into local optimal with imbalanced datasets.

To mitigate the problem of imbalanced credit datasets in unsupervised classification, we introduce a two-stage hybrid system with spectral clustering and semi-supervised SVM (TSC-SVM). This algorithm is designed to address the difficulty in identifying minority-class samples in imbalanced datasets using unsupervised classification algorithms. The original idea is from the attention mechanism. It is expected that the first stage model will assist the second stage model in focusing on certain credit applicants. In the first stage, the dataset is partially labeled using three spectral clustering classifiers based on multiple views which is called the multi-view combined unsupervised method. The multi-view combined unsupervised method exploits complementary information among multiple views based on a probability transfer matrix. Spectral clustering is chosen owing to its ability to yield a computationally optimal solution, thereby enhancing the overall robustness of the model. This stage will help the algorithm estimate the size of different clusters to solve that clustering algorithms create clusters of similar size. The second stage involves a semi-supervised SVM for addressing imbalances in an unsupervised manner by applying varied constraints. Semi-supervised SVMs are a family of techniques for inferring from both labeled and unlabeled data. Unlike existing methods for imbalanced unsupervised classification, the proposed strategy involves segmenting a sample dataset into multiple perspectives using the probability transition matrix. This segmentation significantly enhances the prediction accuracy and robustness of the model. Moreover, by targeting a global optimal solution, the proposed model avoids the pitfall of settling into local optimal. The key contributions to this work can be summarized as follows:

- We introduce the TSC-SVM approach capable of circumventing the challenges typically encountered in unsupervised classification algorithms, such as SVM-based unsupervised methods and spectral clustering, which often classify data into two balanced classes even when the original dataset is imbalanced [21]. The robustness and applicability of the proposed method are validated across various publicly available imbalanced credit datasets.
- We establish a perspective decomposition strategy named the multi-view combined unsupervised method, based on a probability transfer matrix. Using perturbation theory, three different adjacency matrices are constructed from the probability transfer matrix: forward, backward and combined adjacency matrix. This integration enhances the accuracy and robustness of the proposed model through unsupervised classification based on three distinct perspectives.
- We develop a semi-supervised SVM algorithm characterized by strong and weak constraints for labeled and unlabeled samples, respectively. This distinctive approach enables the creation of two hyperplanes that effectively constrain unlabeled samples while mitigating the influence of noise and outlier samples on the separation plane. Consequently, this algorithm can effectively detect and handle imbalanced samples.

The remaining paper is organized as follows: Section 2 provides an overview of unsupervised learning methods and the problem of data imbalance. Section 3 outlines the proposed strategies and their simple reformulation to establish the TSC-SVM approach. Section 4 presents the results of numerical experiments conducted on various imbalanced credit datasets to assess the performance of the proposed method. Section 5 presents the concluding remarks and highlights directions for future research.

## 2 Related work

This section provides a brief review of imbalanced classification methods, hybrid model and unsupervised learning methods.

### 2.1 Imbalanced credit classification

An imbalanced dataset is one in which one or more classes significantly outnumber others [24]. This imbalance is a prevalent challenge in credit risk assessment, where lenders frequently encounter datasets with a large number of repaid loans but few defaults [25]. Machine learning methods face challenges in learning from such imbalanced datasets, as classifiers are inclined to predict predominantly the majority class, reflecting real-world conditions [26]. Three approaches can be used to address the issue of imbalance, i.e., resampling, cost-sensitive learning, and ensemble learning methods [27].

Resampling methods adjust the number of instances in different classes within a dataset, incorporating undersampling and oversampling steps. Undersampling reduces the size of the majority class to balance the dataset. A representative undersampling method is one-sided selection, which identifies and eliminates duplicate or noisy samples from the majority class, thereby reducing its size [28]. In contrast, oversampling addresses class imbalances by augmenting the number of minority-class samples. A well-known example is the synthetic minority oversampling technique (SMOTE), which creates additional samples by interpolating between existing minority-class samples based on spatial similarity [29]. SMOTE has been noted to be more effective than other resampling algorithms in handling imbalanced credit classification problems [30]. Elbow Fuzzy Noise Filtering SMOTE which can effectively reduce the noise within the cluster and thus improve prediction accuracy [31]. The semi-supervised hybrid resampling (SSHR) method which runs semi-supervised clustering to capture the data distribution for both over-sampling and under-sampling [32]. However, a significant limitation of both undersampling and oversampling methods is their reliance on labeled samples, rendering them unsuitable for addressing imbalance in unsupervised learning contexts.

Cost-sensitive methods can be integrated into the learning framework to address problems related to imbalanced sample distributions and the costs associated with misclassification. For example, the cost-sensitive online gradient descent approach, based on a modified hinge loss function, can address the imbalance problem in credit online learning [33]. Additionally, a cost-sensitive SVM has been proposed for generating probabilistic outputs for credit scoring [34]. However, cost-sensitive functions rely on labeled samples and prior information, rendering them more suitable for solving supervised imbalanced credit classification problems, where such samples and knowledge are available.

Ensemble learning methods enhance the accuracy of individual classifiers by amalgamating the decisions of multiple distinct classifiers to produce a singular classification. For instance, multi-layer structured gradient boosted DTs with light gradient boosting machines have been effectively employed to resolve the imbalance problem in peer-to-peer (P2P) lending. However, these approaches require the adjustment of a cost-sensitive function based on labeled data. The cost-sensitive boosted tree method can identify potential default borrowers within labeled P2P datasets [35]. In particular, by leveraging the collective insights of multiple DTs, this method can enhance the predictive accuracy in scenarios characterized by imbalanced class distributions [36]. The Lasso-logistic regression ensemble(LLRE) learning algorithm, an ensemble model based on logistic regression, splits the training data set into several small balanced subsets [37]. BIRCH Clustering Borderline SMOTE (BCBSMOTE) sloved the problem of highly skewed credit card transaction dataset [38]. However, the logistic-BWE algorithm and BCBSMOTE also rely on sample labels for resampling.

Although scholars have extensively explored the issue of imbalanced datasets in credit evaluation, the focus has been on supervised learning contexts. Research on addressing unsupervised imbalanced credit datasets remains limited, despite the considerable attention the imbalanced problem has received in other domains, such as media data and image segmentation. This research gap highlights the need for additional research in the domain of unsupervised classification within credit evaluation, considering the unique challenges and methodologies associated with unsupervised learning entails compared with supervised approaches [21, 22].

### 2.2 Unsupervised learning

Unsupervised learning is distinguished by its ability to make decisions or identify patterns based on unlabeled data [39]. This aspect is particularly advantageous in the context of financial institutions, as it eliminates the need to wait for loan outcomes, thereby facilitating the efficient updating of models [40]. Additionally, the ease of obtaining unlabeled data presents an opportunity for financial institutions to expand their businesses. Because unsupervised learning does not rely on pre-labeled outcomes, it can effectively utilize the vast amounts of readily available, unlabeled financial data, offering a more agile and potentially insightful means of understanding and predicting financial behaviors and trends [10]. Although some studies have semi-supervised methods to solve the problem of lack of sample labels [41], they still cannot solve the problem of credit risk assessment of unlabeled samples.

Traditional unsupervised learning methods, commonly referred to as clustering techniques, can be categorized into prototype-based (using efficient algorithms such as K-means) [16, 42], hierarchical (aimed at discovering nested clusters) [43] density-based (designed to identify regions with varying densities) unsupervised learning [44], and neural networks [45]. Despite their utility, these traditional unsupervised learning algorithms often struggle with sample distributions and are prone to converging on local optima [46]. To enhance the robustness and accuracy of unsupervised learning, SVM-based models, specifically one-class SVMs (OC-SVMs), have been introduced. OC-SVMs cluster data based on a single type of label, offering a novel approach to unsupervised learning [47]. Subsequent developments, such as the weighted one-class SVM and fuzzy one-class SVM, aim to enhance the accuracy and efficiency of OC-SVMs [18]. However, OC-SVM-based methods rely initial labeled data points. To address this limitation, the unsupervised quadratic surface SVM (US-QSSVM) was developed, improving the performance of SVM-based unsupervised learning algorithms on credit risk datasets [10]. Nonetheless, these algorithms are not inherently suitable for handling imbalanced datasets in unsupervised learning scenarios, which represents a crucial concern for credit risk assessment.

In unsupervised imbalanced learning, the majority class often forms clusters, overshadowing the minority class, which may hold greater significance. Consequently, scholars have explored solutions for unsupervised learning in imbalanced datasets. For instance, the golden-rule QSSVM (golden-rule-QSSVM) approach was developed to adjust the separation plane in such scenarios [10]. However, its inability to solve for a global optimal solution limits its accuracy. Another approach is the partition constrained minimum cut (PCut), aimed at finding minimum cut partitions while maintaining minimum cluster sizes [21]. Although PCut can address certain aspects of the imbalanced problem, its computational efficiency is hampered by the complexity involved in searching for optimal parameters. Imbalanced clustering and optimal margin distribution for clustering approaches have been designed for high-dimensional imbalanced data [22, 46]. However, the complexity and specific conditions required for the effective implementation of these algorithms restrict their generalizability in diverse scenarios. Thus, it is necessary to identify robust and versatile unsupervised learning solutions capable of effectively managing imbalanced datasets. Although the isolation forest and copula-based outlier detection method can manage highly imbalanced fraud data [48], its effectiveness on general imbalanced datasets remains questionable.

## 3 Proposed method

In this section, we first introduce the multi-view combined unsupervised method, which leverages multiple perspectives to analyze data, enhancing the ability to uncover hidden patterns and relationships in unlabeled datasets. This approach is particularly effective in addressing the complexities and nuances of imbalanced datasets. Subsequently, we describe the semi-supervised SVM model, designed to bridge the gap between unsupervised and supervised learning by using both labeled and unlabeled data. This model is particularly adept at handling scenarios where labeled data is scarce or incomplete, rendering it valuable for managing imbalanced datasets in a semi-supervised context. These two techniques can be combined to address the challenges posed by imbalanced datasets in various domains.

### 3.1 Multi-view combined unsupervised method

Given the limited accuracy of unsupervised classification algorithms when applied to imbalanced datasets, it is necessary to address the challenge of local optima commonly encountered by traditional unsupervised algorithms, particularly in credit risk assessment. To this end, sufficient information must be mined from data samples. The main purpose of this stage is to identify samples with obvious credit risks. In this study, a bagging strategy is used to address the shortcomings of a single unsupervised classifier. Spectral clustering is adopted as the classifier because it can attain global optimal solutions. In general, spectral clustering algorithms determine the similarity between samples based on a distance-based adjacency matrix. Considering the difficulty of sampling unlabeled datasets, we train different classifiers by constructing different adjacency matrices.

In the first stage, we construct three adjacency matrices for training the ensemble model and labeling certain samples. Consider an unlabeled sample space $\left\{x_{i}\in R^{m}\right\}$ including *n* points. The function *d*(*x*^*i*^, *x*^*j*^) represents the Euclidean distance between any two points *x*^*i*^ and *x*^*j*^. Assuming that the transition probabilities between sample points are related to their Euclidean distances, we then construct a sample-based transition matrix *P* to capture the relationship and transition likelihoods between different points in the sample space, which means we have *n* states in the matrix *P*, providing a foundation for effectively identifying and labeling a portion of the samples based on their relative positions and distances. This approach is particularly valuable for dissecting and understanding the structure of imbalanced datasets, in which traditional methods might overlook subtler yet significant patterns owing to the dominance of the majority class. Thus, by breaking down the sample space into multiple perspectives and examining the relationships between points, a more nuanced and robust understanding of the dataset can be achieved, leading to improved unsupervised classification in credit risk assessment. In other words, identifying borrowers with obvious credit risk, whether they’re extremely low or extremely high default risks, is easy with any distant measurement method. Therefore, the multi-view method can efficiently select some samples with obvious credit risks, which will be beneficial for the model to estimate the imbalance ratio of samples.

$$\begin{array}{c} p_{ij}=\frac{\text{exp}(-\frac{d_{ij}}{2})}{\sum_{j=1}^{n}\text{exp}\left(-\frac{d_{ij}}{2}\right)}, \end{array}$$

$$\begin{array}{c} P\;=\;[\begin{array}{cccc} p_{11} & p_{12} & \ldots & p_{1n}\\ p_{21} & p_{22} & \ldots & p_{2n}\\ \vdots & \vdots & \ddots & \vdots \\ p_{n1} & p_{n2} & \ldots & p_{nn} \end{array}] \end{array}$$

In this model, each element *p*_*ij*_ of the transition matrix *P* is defined independent of the parameters of the radial basis function. This setting helps enhance the model robustness by eliminating the need for parameter selection. *p*_*ij*_ represents the probability that sample point *i* is transformed into sample point *j*. In other words, *p*_*ij*_ is an asymmetric measure of the correlation between sample points *i* and *j*, as *p*_*ij*_ is not equal to *p*_*ji*_, unlike the traditional distance-based symmetry measure. Spectral clustering is an unsupervised learning method based on the relationship between sample points. The objective is to ensure that samples within the same cluster are as similar as possible, while those in different clusters are distinct [49].

To more effectively extract information from the samples and enhance the clustering efficiency, we decompose the probability transfer matrix *P* into two weighted adjacency matrices based on the directional differences in sample transitions. Specifically, the probability of sample *x*_*i*_ transitioning to *x*_*j*_ is not necessarily the same as that of *x*_*j*_ transitioning to *x*_*i*_. Consequently, we split matrix *P* into a forward adjacency matrix *F* (representing the forward view) and backward adjacency matrix *B* (representing the backward view), each corresponding to the direction of sample transformation. By considering both forward and backward transition probabilities, we can better capture the asymmetric nature of the relationships within the dataset, leading to more accurate and effective clustering, particularly in imbalanced datasets commonly encountered in credit risk assessment.

$$\begin{array}{c} F\;=\;[\begin{array}{cccc} p_{11} & p_{12} & \ldots & p_{1n}\\ p_{11} & p_{22} & \ldots & p_{2n}\\ \vdots & \vdots & \ddots & \vdots \\ p_{1n} & p_{2n} & \ldots & p_{nn} \end{array}] \end{array}$$

$$\begin{array}{c} B\;=\;[\begin{array}{cccc} p_{11} & p_{21} & \ldots & p_{n1}\\ p_{21} & p_{22} & \ldots & p_{n2}\\ \vdots & \vdots & \ddots & \vdots \\ p_{n1} & p_{n2} & \ldots & p_{nn} \end{array}] \end{array}$$

Matrices *F* and *B* can be verified to be symmetric and positive semi-definite. However, these matrices are sensitive to the order in which the samples are input. To mitigate the influence of sample order on the results of unsupervised learning, we propose a strategy to sort the samples based on the distance *d*(*x*_*i*_, *o*) from their positions *x*_*i*_ to a central point $o\in R^{m}$, typically defined as the centroid or mean of the sample space. This sorting in descending order ensures that the sample order does not unduly influence the learning process. Subsequently, we define two normalized graph Laplacians matrices, which are crucial to the spectral clustering process as they encapsulate the essential structure of the data by reflecting the relationships and connections between different samples. The normalization of these matrices ensures that the clustering process is not disproportionately influenced by variations in sample size or density, yielding a more balanced and accurate representation of the underlying data structure. This aspect is particularly beneficial in the context of imbalanced datasets, where traditional methods may be skewed by the dominance of the majority class.

$$\begin{array}{c} L_{F}=D_{F}^{-1/2}FD_{F}^{-1/2}, \end{array}$$

$$\begin{array}{c} L_{B}=D_{B}^{-1/2}FD_{B}^{-1/2}, \end{array}$$

The degree matrix *D* is a key component in defining the normalized graph Laplacians matrices. Specifically, *D* is a diagonal matrix where each element on the diagonal, *d*_1_, …, *d*_*n*_, represents the degree of a node in the graph. The node degree typically refers to the sum of the weights of the edges connected to that node. In spectral clustering, these weights are derived from the adjacency matrices *F* and *B*. To enhance the accuracy of the proposed algorithm, especially in handling imbalanced datasets, we introduce a third perspective through weighted adjacency matrices. In particular, we define a combination matrix *C*, which merges the information encapsulated in both *F* and *B*. *C* is designed to synthesize the distinct but complementary perspectives provided by the forward and backward adjacency matrices, offering a more comprehensive view of the relationships and structures within the dataset. By integrating this third perspective, a more complete understanding of the data can be achieved, which is crucial in unsupervised learning scenarios.

$$\begin{array}{c} L_{C}=\mu_{1}L_{F}+\mu_{2}L_{B}, \end{array}$$

In constructing the combination matrix *C*, we use real non-negative numbers *μ*_*i*_, where *μ*_1_ + *μ*_2_ = 1. This ensures that the combined matrix is a weighted average of the forward and backward perspectives, balancing the influence of each. The normalized graph Laplacians matrix for this combined view can then be defined as *L*_*C*_ = *μ*_1_*L*_*F*_ + *μ*_2_*L*_*B*_. To ensure coherence and avoid excessive discrepancies between clustering results under the three perspectives (forward, backward, and combined), we seek an intermediate perspective that balances the forward and reverse views. To this end, we introduce the canonical angle, which quantifies the difference in clustering ability between the eigenvectors of the forward and backward views [50]. This metric is particularly useful for understanding how different perspectives may influence the final clustering results and for adjusting weights *μ*_1_ and *μ*_2_ accordingly to achieve an optimal balance. By carefully calibrating these weights based on the canonical angle, the clustering performance of the proposed algorithm can be enhanced, especially in scenarios involving imbalanced datasets.

**Definition 1**. Let *V*_*k*_ and $\tilde{V_{k}}$ be subspaces spanned by the orthonormal eigenvectors *v*_*i*_, …, *v*_*i*+*k*_ and $\tilde{v_{i}},\cdots ,\tilde{v_{i+k}}$, respectively. Moreover, let *γ*_1_ ≤ … ≤ *γ*_*k*_ represent the singular values of ${\left(v_{i},\cdots ,v_{i+k}\right)}^{T}\left(\tilde{v_{i}},\cdots ,\tilde{v_{i+k}}\right)$. Then,

$$\begin{array}{c} \theta_{i}={\text{cos}}^{-1}\gamma_{i}, \end{array}$$ (1)

where *θ*_*i*_ represents the canonical angles between *V*_*k*_ and $\tilde{V_{k}}$.

If the largest canonical angle, defined in Eq (1), is small, the eigenvectors *V*_*k*_ and $\tilde{V_{k}}$ are closely aligned. This observation is significant in spectral clustering, where clustering results depend on the first *k* eigenvectors of the Laplacian matrix. Consequently, a critical task in enhancing the clustering process is to find the optimal pair of weights (*μ*_1_, *μ*_2_). These weights should be chosen such that the first *k* eigenvectors of the Laplacian matrix *L*_*C*_ closely approximate the *k* eigenvectors $V_{k}^{F}$ and $V_{k}^{B}$ of the Laplacian matrices *L*_*F*_ and *L*_*B*_, respectively. To accomplish this, we introduce a theorem related to the canonical angle to quantitatively assess the closeness of these eigenvectors [50] and determine *μ*_1_ and *μ*_2_ that result in the most effective clustering by leveraging the combined strengths of both forward and backward views. This selection ensures that the combined view represented by *L*_*C*_ effectively merges the information encapsulated in both *L*_*F*_ and *L*_*B*_, leading to a more accurate and robust clustering outcome, especially in the context of imbalanced datasets.

**Theorem 3.1** Let $\lambda_{i},v_{i},\tilde{\lambda_{i}}$, and $\tilde{v_{i}}$ represent the *i*^*th*^ eigenvalues and eigenvectors of *L* and $\tilde{L}$, respectively. Additionally, let *Θ* = *diag*(*θ*_1_, …, *θ*_*k*_) be the diagonal matrix of canonical angles between the column space of *V*_*k*_ = (*v*_1_, …, *v*_*k*_) and $\tilde{V_{k}}=\left(\tilde{v_{1}},\cdots ,\tilde{v_{k}}\right)$. If there exists a gap *η* > 0 such that $|\tilde{\lambda_{k}}-\lambda_{k+1}|\geq \eta$ and $\tilde{\lambda_{k}}\leq \eta$, then

$$\begin{array}{c} {\left||\text{sin}\Theta |\right|}_{F}\leq \frac{1}{\eta }||L\tilde{V_{k}}-\tilde{V_{k}}\tilde{\Sigma_{k}}{\left|\right|}_{F}, \end{array}$$ (2)

where sin Θ is defined entry-wise, and $\tilde{\Sigma_{k}}=diag\left(\tilde{\lambda_{1}},\cdots ,\tilde{\lambda_{k}}\right)$.

Following the literature, the gap *η* can be considered a constant approximating the ground truth Laplacian matrix *L* [51]. Therefore, we can minimize the upper bound of Eq (2) to obtain the closest eigenvectors $V_{k}^{C}$ to $V_{k}^{F}$ and $V_{k}^{B}$. The optimization function is defined as

$$\begin{array}{cc} \underset{\mu_{1},\mu_{2}}{\text{min}}\; & \left|\right|L^{C}\tilde{V_{k}^{F}}-\tilde{V_{k}^{F}}\tilde{\Sigma_{k}^{F}}{\left|\right|}_{F}^{2}+\left|\right|L^{C}\tilde{V_{k}^{B}}-\tilde{V_{k}^{B}}\tilde{\Sigma_{k}^{B}}{\left|\right|}_{F}^{2},\\ s.t.\; & \mu_{i}\geq 0,\;i=1,2,\\ & \mu_{1}+\mu_{2}=1. \end{array}$$ (3)

Clearly, Eq (3) represents a quadratic programming problem, and thus, the optimal $\left(\mu_{1}^{*},\mu_{2}^{*}\right)$ can be determined from the optimal solution of Eq (3). We perform spectral clustering on the three Laplacian matrices *L*^*F*^, *L*^*B*^, and *L*^*C*^, obtaining results $y_{i}^{F}\in \left\{-1,1\right\}$, $y_{i}^{B}\in \left\{-1,1\right\}$, and $y_{i}^{C}\in \left\{-1,1\right\}$, respectively. Details of the spectral clustering algorithm are not presented herein for simplicity and can be found elsewhere [52]. The samples are labeled *y*_*i*_ using a one-vote veto strategy to obtain the most robust results:

$$\begin{array}{c} y_{i}=y_{i}^{C}\;\text{if}y_{i}^{F}=y_{i}^{B}\;and\;y_{i}^{F}=y_{i}^{C}. \end{array}$$

The multi-view combined unsupervised method presents two advantages for unsupervised imbalanced learning: First, all results are obtained using the global optimal solution. Second, the number of parameters to be selected is reduced. These aspects help enhance the robustness and generalizability of the proposed method. This stage will help the algorithm estimate the size of different clusters to solve that clustering algorithms create clusters of similar size. In other words, identify applicants with evident credit risk characteristics at this stage, regardless of whether they are good or bad. This will help the subsequent algorithm to determine the size of different clusters by separating the planes.

**Algorithms 1:** Multi-view combined unsupervised method

1: **Input:** data point: *x*_*i*_

2: Calculate the central point ← *x*_*i*_

3: Sort the data point $x_{i}^{*}$ by the *distance*(*x*_*i*_, *o*)

4: Calculate forwoad and backward normalized graph Laplacians matrix *L*_*F*_ and *L*_*B*_ ← $x_{i}^{*}$

5: Calculate the adjusting weights *μ*_1_ and *μ*_2_ by solved quadratic programming problem Eq (3)

6: Calculate *L*_*C*_ ← *μ*_1_*L*_*F*_ + *μ*_2_*L*_*B*_

7: Obtain the label $y_{i}^{F}\in \left\{-1,1\right\}$, $y_{i}^{B}\in \left\{-1,1\right\}$, and $y_{i}^{C}\in \left\{-1,1\right\}$ by the spectral clustering with three Laplacian matrices *L*^*F*^, *L*^*B*^, and *L*^*C*^

8: Define the labled *y*_*i*_ using a one-vote veto strategy with the label $y_{i}^{F}\in \left\{-1,1\right\}$, $y_{i}^{B}\in \left\{-1,1\right\}$, and $y_{i}^{C}\in \left\{-1,1\right\}$

9: **Output:** labeled *y*_*i*_

### 3.2 Semi-supervised SVM

**Fig 1:**
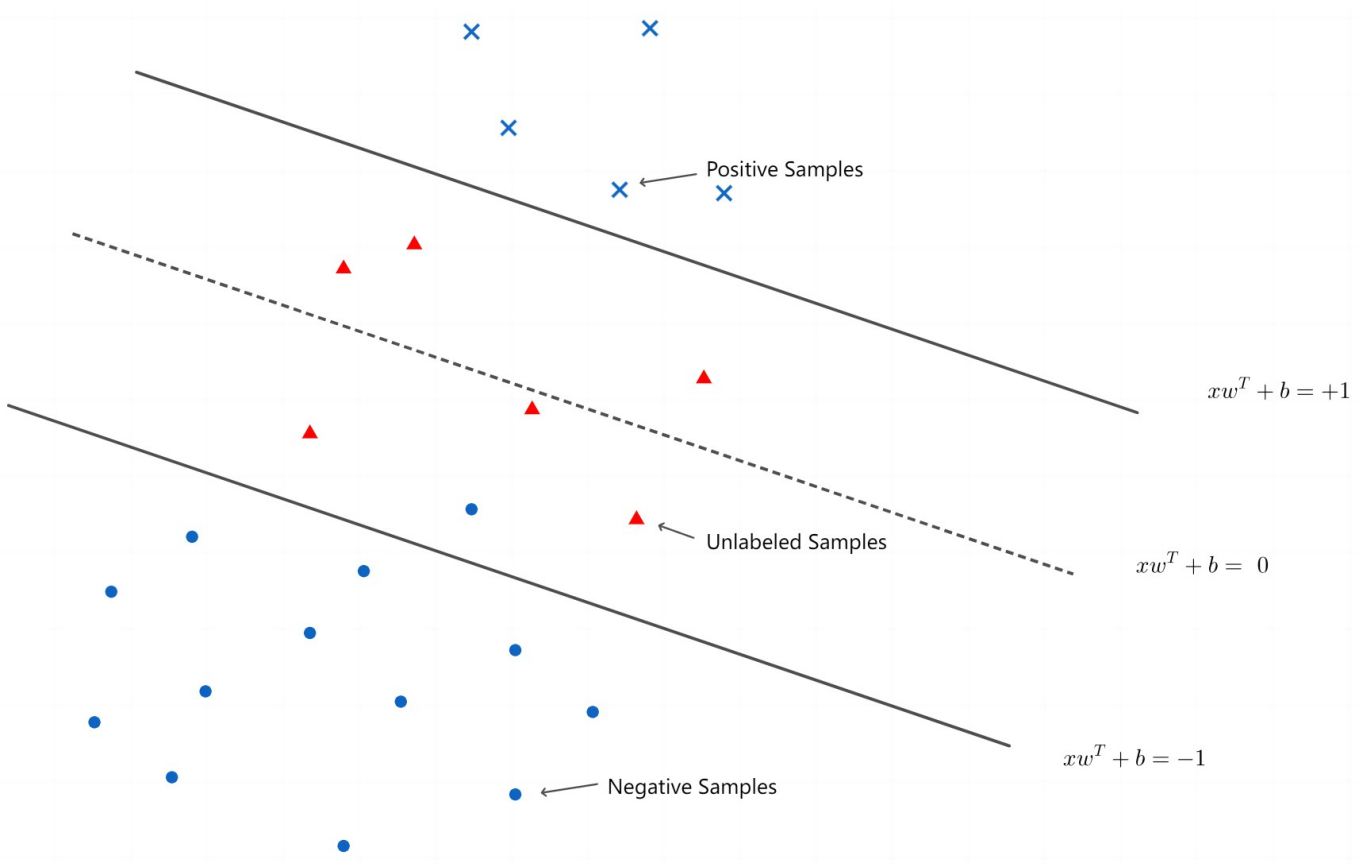
Framework of the semi-supervised support vector machine.

In stage 1, when $y_{i}=y_{i}^{C}$, indicating consensus among several classifiers, we can effectively partition the sample set into two groups. The first group consists of labeled samples, denoted as $\left\{a_{i}\in R^{m}\right\}\subset \left\{x_{i}\right\}$, each with an associated label *y*_*i*_. The second group includes unlabeled samples, represented as $\left\{q_{i}\in R^{m}\right\}\subset \left\{x_{i}\right\}$. To facilitate this division, we define a matrix $A\in R^{n_{1}*m}$ for the labeled samples, which consists of vector *a*_*i*_, where *i* = 1, …, *n*_1_. Similarly, the matrix $Q\in R^{n_{2}*m}$ for the unlabeled samples consists of vector *q*_*i*_, where *i* = 1, …, *n*_2_. This approach is inspired by the principles of SVM-based unsupervised methods. The core concept of the semi-supervised SVM model is to position all labeled points outside two hyperplanes defined by the equations *xω*^*T*^ − *b* = 1 and *xω*^*T*^ − *b* = −1. Simultaneously, the model aims to confine the unlabeled samples between these two hyperplanes as tightly as possible, ensuring clear and effective separation of classes. This configuration is illustrated in Fig 1, where red and blue dots represent unlabeled samples {*q*_*i*_} and labeled samples {*a*_*i*_}, respectively. The two hyperplanes *xω*^*T*^ − *b* = 1 and *xω*^*T*^ − *b* = −1 constrain the labeled sample points on the outside and unlabeled sample points on the inside.

In the semi-supervised SVM framework, the objective is to maximize the margin, represented as 2/||*ω*||, while adhering to specific constraints. These constraints ensure that all unlabeled points lie between the two hyperplanes defined by *xω*^*T*^ + *b* = 1 and *xω*^*T*^ + *b* = −1, and that all labeled samples are situated outside these hyperplanes. To introduce flexibility and manage potential misclassification or overlap, slack variables $\xi =(\xi_{1},\cdots ,\xi_{n_{1}})$ and $\sigma =(\sigma_{1},\cdots ,\sigma_{n_{1}})$ are incorporated into the model. These variables allow for a relaxation of the constraints, accommodating instances where it may not be feasible to strictly enforce the original conditions. Consequently, the semi-supervised model with these slack variables is formulated as follows:

$$\begin{array}{cc} \text{min}\; & \frac{1}{2}{\left||\omega |\right|}^{2}+C_{1}\sum_{i=1}^{n_{1}}\xi_{i}+C_{2}\sum_{i=1}^{n_{2}}\sigma_{i},\\ s.t.\; & y(A\omega^{T}+b)\geq 1-\xi ,\\ & |Q\omega^{T}+b|\leq 1+\sigma ,\\ & \xi \geq 0,\;\sigma \geq 0. \end{array}$$ (4)

In this model, *C*_1_ and *C*_2_ are penalty coefficients, which determine the strength of constraints on labeled and unlabeled samples. Because labeled samples clearly indicate the credit risk, strong and weak constraints are imposed on labeled and unlabeled samples, respectively. In other words, *C*_1_ ≫ *C*_2_. Thus, the formulation provided in Eq (4) constitutes a convex optimization problem. The convexity of this problem is advantageous, ensuring that any local optimum found is also a global optimum. To efficiently solve this optimization problem, a common approach is to derive the dual problem. The dual formulation of an optimization problem often simplifies the solution process, particularly in the context of SVMs. The Lagrangian function, presented in Eq (5), is formulated using Lagrange multipliers *β*^1^, *β*^2^, *β*^3^, *β*^4^, *β*^5^ ≥ 0. These multipliers facilitate the relaxation of the primal constraints and enable the transition from the primal to the dual problem. To identify the saddle point of the Lagrangian function, which corresponds to the optimal solution of the problem, the partial derivatives of the Lagrangian function with respect to the primal variables (*ω*, *b*, *ξ*, *σ*) are determined. Specifically, these derivatives are set to zero, and the resulting equations are solved, a standard procedure in convex optimization.

$$\begin{array}{cc} L_{SVM}= & \frac{1}{2}{\left||\omega |\right|}^{2}+C_{1}\sum_{i=1}^{n_{1}}\xi_{i}+C_{2}\sum_{i=1}^{n_{2}}\sigma_{i}\\ & +\sum_{i=1}^{n_{1}}\beta_{i}^{1}\left(1-\xi_{i}-y_{i}\left(a_{i}\omega^{T}+b\right)\right)-\sum_{i=1}^{n_{1}}\beta_{i}^{2}\xi_{i}\\ & -\sum_{i=1}^{n_{2}}\beta_{i}^{3}\left(1+\sigma_{i}-\left(q_{i}\omega^{T}+b\right)\right)-\sum_{i=1}^{n_{2}}\beta_{i}^{4}\left(1+\sigma_{i}+\left(q_{i}\omega^{T}+b\right)\right)\\ & -\sum_{i=1}^{n_{2}}\beta_{i}^{5}\sigma_{i}, \end{array}$$ (5)

Suppose the partial derivates of *L*_*SVM*_ with respect to the primal variables (*ω*, *b*, *ξ*, *σ*) equal zero. Then, we can obtain the conditions for optimality corresponding to the following dual problem:

$$\begin{array}{cc} \text{max}\; & \sum_{i=1}^{n_{1}}\beta_{i}^{1}-\sum_{i=1}^{n_{2}}\left(\beta_{i}^{3}+\beta_{i}^{4}\right)-\frac{1}{2}{\left(\sum_{i=1}^{n_{1}}\beta_{i}^{1}y_{i}a_{i}+\sum_{i=1}^{n_{2}}\left(\beta_{i}^{4}-\beta_{i}^{3}\right)q_{i}\right)}^{2},\\ s.t.\; & \sum_{i=1}^{n_{1}}\beta_{i}^{1}y_{i}+\sum_{i=1}^{n_{2}}\left(\beta_{i}^{4}-\beta_{i}^{3}\right)=0,\\ & 0\leq \beta_{i}^{1}\leq C_{1},\;0\leq \beta_{i}^{2}\leq C_{1},\\ & 0\leq \beta_{i}^{3}\leq C_{2},\;0\leq \beta_{i}^{4}\leq C_{2},\;0\leq \beta_{i}^{5}\leq C_{2}. \end{array}$$ (6)

For the sake of brevity and clarity, detailed mathematical derivations and the Karush–Kuhn–Tucker (KKT) conditions relevant to the semi-supervised SVM are presented in S1 Appendix. Overall, the optimal solution for the semi-supervised SVM can be obtained using quadratic optimization methods, as outlined in Eq (6). Quadratic optimization is a well-established method in optimization theory, particularly suited for SVM frameworks where the objective function and constraints can be expressed in quadratic forms. Once the optimal solution has been determined through this process, it can be used to predict the final classification results within the TSC-SVM. This system integrates the strengths of spectral clustering and semi-supervised learning to effectively handle the challenges posed by imbalanced datasets in unsupervised learning.

**Fig 2:**
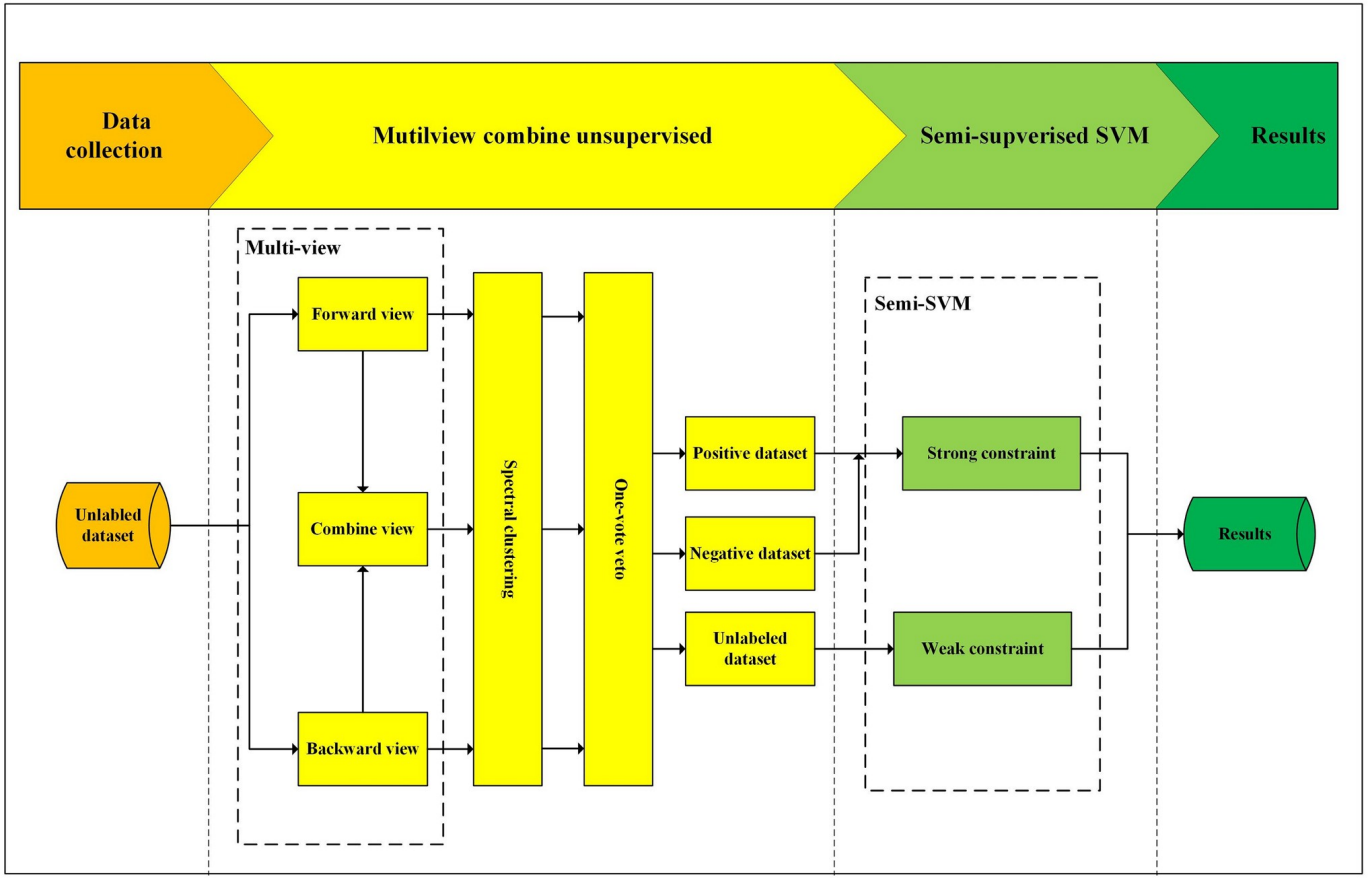
TSC-SVM framework.

Fig 2 shows the TSC-SVM framework. Generally, the TSC-SVM framework selects samples that have obvious credit risks in the first stage and focuses on the remaining samples in the second stage. In the first stage, easy-to-identify samples are labeled using a bagging ensemble method, i.e., the multi-view combined unsupervised method. These labeled samples aid the semi-supervised SVM in determining the optimal separation plane. This stage will help the algorithm estimate the size of different clusters to solve that clustering algorithms create clusters of similar size. It is similar to an attention mechanism, which enables the second-stage model to focus on sample points that are closer to the separation plane. This ensemble learning method overcomes the limitations associated with a single classifier in identifying minority-class samples. The spectral clustering algorithm divides samples into two parts with the highest difference. This will enable samples with obvious credit risks, whether they are “too good” or “too bad” to be identified.

In the second stage, the results of the first stage are used to split the dataset {*x*_*i*_} into two subsets: {*a*_*i*_} (labeled dataset) and {*q*_*i*_} (unlabeled dataset). In semi-supervised SVMs, hyperplanes *xω*^*T*^ + *b* = 1 and *xω*^*T*^ + *b* = −1 aim to maximize the support vectors to separate labeled samples {*a*_*i*_}. In addition, hyperplanes constrain unlabeled samples {*q*_*i*_}, allowing the separation plane *xω*^*T*^ + *b* = 0 to efficiently identify minority-class samples. It should be noted that in the second stage, more focus will be placed on unlabeled samples. Therefore, the semi-supervised support vector machine model will help us achieve this goal and improve model prediction accuracy. Furthermore, spectral clustering and semi-supervised SVM have lower parameter requirements, which is a key reason for their selection as the main classification methods.

**Algorithms 2:** Semi-supervised SVM

1: **Input:** labeled group: $\left\{a_{i}\in R^{m}\right\}\subset \left\{x_{i}\right\}$ with label *y*_*i*_, unlabeled group: $\left\{q_{i}\in R^{m}\right\}\subset \left\{x_{i}\right\}$, penalty coefficients: *C*_1_ and *C*_2_

2: Calculate the *ω* and *b* ← convex programming problem *x*_*i*_ Eq (4)

3: Obatain the label of the data point *x*_*i*_ by the hyperplane *xω*^*T*^ + *b* = 0

4: **Output:** the label of the data point *x*_*i*_

Overall, the TSC-SVM which is expected from the first stage model will assist the second stage model in focusing on certain credit applicants aims to solve the credit assessment in unsupervised imbalanced learning. In the next section, this paper will demonstrate the effectiveness of this method in numerical experiments.

## 4 Results

### 4.1 Experimental setup

To assess the effectiveness of the TSC-SVM model, various public benchmark datasets specific to credit risk were employed. These datasets provided a comprehensive platform for testing and validating the model performance in real-world scenarios. The performance of the TSC-SVM model was compared with those of state-of-the-art methods, including K-means, US-QSSVM, US-QSSVM-GOLD, PCut, spectral clustering algorithm and the unsupervised LDA (Un-LDA) [10, 16, 19, 21, 53]. Testing these methods on the same benchmark datasets enabled a comprehensive comparison across different dimensions, such as accuracy, robustness, and ability to handle imbalanced datasets, thereby clarifying the strengths and limitations of the TSC-SVM model in credit risk assessment compared with existing techniques.

To ensure a fair and thorough evaluation of the TSC-SVM model in real-world unsupervised imbalanced credit classification scenarios, it was tested on entire datasets using specific parameters. There is a lack of labeled samples for optimizing parameters in real unsupervised credit risk classification. Accordingly, the proposed method in this paper will be tested on a variety of datasets under fixed parameter conditions. The *k*-NN weighted adjacency matrix has been proven to be effective in imbalanced datasets [21]. Hence, considering the task to be a binary classification problem, we set *k* = *n*/2 in the experiments, where *n* is the number of samples in the dataset. In the first stage of the TSC-SVM model, different penalty parameters were assigned to labeled (*C*_1_ = 2^10^) and unlabeled samples (*C*_2_ = 1) to account for their distinct roles in the model. This differentiation ensured that labeled samples adhered strictly to the two planes, while allowing some flexibility for unlabeled samples to cross the planes as needed. This approach helped stabilize the separation plane and yield more robust prediction results. To ensure that the experimental results of the TSC-SVM model were not influenced by parameter selection and recognizing that financial institutions may not always have the necessary data to fine-tune parameters, a systematic approach was adopted for parameter selection: For the US-QSSVM and US-QSSVM-GOLD models, the optimal hyper-parameter $\hat{\eta_{1}}$ was determined using a grid method with $log_{2}\hat{\eta_{1}}$ in the range {16, 17, …, 30}, as suggested in the literature [10]. We randomly selected 50% of the original dataset for tuning parameters, repeating this process ten times to ensure statistical significance. The final parameters were chosen based on the highest average accuracy achieved. For the PCut method, the parameters suggested in a prior study were used: [21]: *k*_0_ = 30, $\sigma =\tilde{d_{30}}$, and *δ* = 0.05. Following the literature, the subspace dimension size *m* for Un-LDA is define as *min*{*d*, *c* − 1} where *d* is the number of dimensions and *c* is the number of classes. In all experiments, datasets were processed without labels for training and tested with actual labels for validation. Each reported result represents an average over 20 experiments, ensuring the reliability and validity of the findings. All the experiments are performed on a desktop computer with Intel i9 CPU, 64GB RAM, and Windows 10 operating system.

For validation, four widely recognized public credit benchmark datasets were chosen from the UCI public database and Kaggle [54, 55]. The datasets were selected to ensure that they adequately represented the considered algorithms across a range of conditions and applications. These datasets have been frequently used in various studies focusing on credit risk assessment [35, 56]:

- Australian Dataset (from UCI Database): This balanced credit dataset is selected to test the robustness of the models in a balanced scenario, providing a contrast to the imbalanced datasets typically encountered in credit risk assessment.
- Lending Club Datasets (from Kaggle): The 2018 Q4 (2018Q4) and 2018 Q3 (2018Q3) versions were chosen owing to their relevance to current credit risk evaluation scenarios. These datasets include loans categorized as non-default and late (31–120 days), according to the latest sample available on Kaggle. These datasets offer a real-world context for testing the model performance in handling various loan statuses. In particular, these datasets are selected as they include real imbalanced credit reports provided by financial institutions. Notably, Lending Club is the largest P2P platform in the United States and these datasets have been cited in various credit classification studies.
- Credit Risk Dataset (from Kaggle): This dataset provides additional insights into credit risk assessment, complementing the Lending Club and Australian datasets.

Table 1 provides an overview of these datasets, including the number of samples, distribution of classes, and other relevant characteristics. This information is vital for understanding the context in which the algorithms were tested and interpreting the results of the experiments. The diversity and representativeness of these datasets ensure that the findings of this study are robust and applicable to a broad range of scenarios in the field of credit risk assessment. This article deletes the missing values in the datasets. The experiments use all samples in the dataset without the labels.

**Table 1:** Dataset description.

Dataset	Instances	# Negative	# Positive	IR	Attributes
2018 Q4	13268	11263	2005	5.6	88
2018 Q3	18819	16545	2274	7.3	88
Credit risk	28638	22435	6203	3.6	11
Australian	690	383	307	1.2	14

In evaluating the performance of the proposed model, particularly in the context of imbalanced data distributions commonly encountered in credit datasets, relying solely on the overall accuracy can be misleading. This is because the accuracy metric does not differentiate between the classification performance on the minority class (often more critical in credit risk scenarios) and majority class. For instance, in a dataset with a 90% imbalance ratio, classifying all samples as the majority class would still yield a 90% accuracy rate, despite failing to identify any of the minority-class samples. To more comprehensively evaluate the classifier ability to accurately identify both positive (minority) and negative (majority) classes, four key metrics were used. Table 2 presents a confusion matrix, providing a better understanding of these metrics. The confusion matrix specifies the true positives (TP), true negatives (TN), false positives (FP), and false negatives (FN), which are the basic elements required to compute these metrics. These evaluation metrics were used to enable a balanced and nuanced assessment of the model performance, acknowledging the complexities inherent in imbalanced datasets. Such an approach is critical in demonstrating the efficacy of the proposed model in real-world credit risk assessment scenarios.

**Table 2:** Confusion matrix.

		Predicted Label	
		Positive	Negative
Actual label	Positive	TP	FN
	Negative	FP	TN

The Table 2 shows the definition of TP, FP, TN and FN which illustrates the confusion matrix used for evaluating these metrics, with the minority class designated as the positive class. In credit classification, FN indicates that loan defaulters are predicted to be non-defaulters, leading to substantial economic losses for financial institutions. FP indicates that the classification algorithm predicts non-defaulters as loan defaulters, resulting in the loss of potential customers. The economic loss caused by FN is typically greater than that caused by FP, which is why we emphasize the identification efficiency of the positive class (minority). Based on these definitions, the following three evaluation metrics can be better interpreted:

$$\begin{array}{c} Recall=\frac{TP}{TP+FN} \end{array}$$

$$\begin{array}{c} Precision=\frac{TP}{TP+FP} \end{array}$$

$$\begin{array}{c} F1-measure=2\times \frac{Recall\times Precision}{Recall+Precision} \end{array}$$

These evaluation metrics will help us fairly measure the experimental results with different models. The experimental results will be reported with these indicators.

### 4.2 Experiments results

Table 3 presents the evaluation metrics of the proposed TSC-SVM method as well as the comparative algorithms (K-means, US-QSSVM, US-QSSVM-GOLD, PCut, spectral clustering, and Un-LDA). In comparison against other algorithms across various credit datasets, TSC-SVM consistently performs better, highlighting its robustness and reliability for credit risk assessment in diverse scenarios, particularly those involving imbalanced datasets. This experiment also presents the Wilcoxon test results for the algorithm proposed in this paper and other algorithms that use the F1 indicators in order to support the conclusions of the experiment. ** and * represents the *p*-value is less than 0.01 and 0.1.

**Table 3:** Comparative analysis over various datasets.

		Accuracy	Precision	Recall	F1-Measure
2018 Q4	K-means	0.6746	0.2373	0.2942	0.2627**
	SC	0.5621	0.1643	0.3606	0.2258**
	US-QSSVM	0.6218	0.1622	0.1781	0.1698**
	US-QSSVM-GOLD	0.6151	0.1528	0.1998	0.1732**
	Un-LDA	0.7467	0.5388	0.3486	0.3585*
	PCut	0.6913	0.1458	0.0382	0.0605**
	TSC-SVM	0.6854	0.2737	0.6544	0.3860
2018 Q3	K-means	0.6971	0.1325	0.1612	0.1454**
	SC	0.5688	0.1432	0.2475	0.1815**
	US-QSSVM	0.6235	0.1456	0.2121	0.1727**
	US-QSSVM-GOLD	0.6143	0.1242	0.2311	0.1616**
	Un-LDA	0.5138	0.1317	0.3732	0.1695**
	PCut	0.7188	0.1109	0.1891	0.1398**
	TSC-SVM	0.6348	0.1740	0.5395	0.2631
Credit risk	K-means	0.6069	0.2895	0.3003	0.2948**
	SC	0.5418	0.1983	0.1916	0.1949**
	US-QSSVM	0.6506	0.3010	0.2708	0.2851**
	US-QSSVM-GOLD	0.5870	0.2597	0.1985	0.2250**
	Un-LDA	0.5346	0.2778	0.5136	0.3333**
	PCut	0.7617	0.4213	0.2685	0.3280**
	TSC-SVM	0.6939	0.3738	0.6116	0.4640
Australian	K-means	0.7200	0.6390	0.6161	0.6274**
	SC	0.7891	0.7091	0.7924	0.7484**
	US-QSSVM	0.5675	0.4675	0.4442	0.4556**
	US-QSSVM-GOLD	0.5684	0.4471	0.4471	0.4478**
	Un-LDA	0.7344	0.6505	0.6723	0.6535**
	PCut	0.7920	0.8228	0.5657	0.6704**
	TSC-SVM	0.8397	0.8432	0.7340	0.7848**

Specifically, the results in 2018 Q4, 2018 Q3 and credit risk datasets show that the TSC-SVM algorithm demonstrates enhanced capability in recognizing the minority class, which is crucial in credit risk assessment where identifying potential defaulters is more critical than identifying reliable borrowers. This improvement can potentially help financial institutions reduce losses due to credit defaults. Although the PCut algorithm exhibits higher accuracy in certain datasets (2018 Q4, 2018 Q3, and Credit Risk), it cannot effectively identify credit defaulters in imbalanced datasets. For the balanced dataset Australian, the TSC-SVM model also improves the identification of high-risk credit cases. Such a method is invaluable for financial institutions that require accurate and reliable models for risk assessment to manage and mitigate credit risk. For example, the increase in the recall indicator means that more loan defaulters are accurately identified, preventing financial institutions from suffering significant losses owing to loan defaults, as in the case of 2018 Q4 and 2018 Q3.

The experimental results of all algorithms on balanced datasets are better than those on imbalanced datasets including 2018 Q4, 2018 Q3 and credit risk datasets. This means that the imbalance problem has a negative impact on unsupervised algorithms. There are two main reasons. First, clustering algorithms create clusters of similar size. This means that traditional unsupervised algorithms ignore the imbalanced proportion of data. Certain algorithms, such as US-QSSVM and spectral clustering, inherently divide the dataset into two balanced categories based on their optimization functions. This approach fails to account for the inherent imbalance in the data, thereby neglecting the minority class, which is often of greater interest in credit risk assessment. Although the PCut algorithm is designed to address imbalanced problems in an unsupervised manner, it has its own set of challenges. The complex parameter search mechanism required for PCut leads to high computational complexity and reduced efficiency. Then, prior knowledge is required to set parameters such as *k* and *δ*, undermining the generalization capability and robustness of the algorithm. These issues limit the practical applicability of PCut in diverse real-world scenarios, particularly in financial contexts where data may not always conform to predefined assumptions. Second, in the absence of prior knowledge, traditional unsupervised learning algorithms are prone to converging on local optimal solutions. This issue becomes more pronounced in imbalanced datasets where the minority class is underrepresented, leading to biased or incomplete learning outcomes. These insights underscore the need for more sophisticated and tailored approaches, such as the TSC-SVM model, designed to address the challenges of unsupervised imbalanced learning in credit risk assessment. The three parameters that need to be determined for this model include *k*, *C*_1_, and *C*_2_. Penalty parameters *C*_1_ and *C*_2_ are used to strengthen labeled sample constraints, and thus, *C*_1_ must be sufficiently large. The parameters *k*, *C*_1_ and *C*_2_ are discussed in more detail in a later section. Such methods are crucial for financial institutions seeking reliable and efficient tools for risk evaluation in the increasingly complex and data-driven financial landscape.

Notably, the proposed algorithm demonstrates robust and efficient performance across both balanced and imbalanced datasets, a significant achievement considering the distinct challenges posed by each type of dataset. The effectiveness of our algorithm does not rely on prior knowledge for parameter adjustment, which is a common limitation in many traditional models. Its ability to efficiently classify all credit datasets, even with fixed parameters, highlights its adaptability and ease of use. The robustness and accuracy of the proposed algorithm across a diverse range of datasets can be attributed to two key factors: The TSC-SVM approach is designed to derive the global optimal solution at all steps of the process, effectively circumventing the issue of local optimal solutions. This global perspective ensures that the algorithm comprehensively considers the entire dataset, leading to more accurate and reliable outcomes. This method provides stable and accurately labeled samples, which are crucial for the effective performance of the semi-supervised SVM. Second, by incorporating multiple views, the algorithm can capture a broader and more nuanced understanding of the data, thereby enhancing the quality of the labeled samples and optimizing the separation plane of the semi-supervised SVM, further improving its classification capabilities. Overall, the favorable performance of the proposed algorithm across both balanced and imbalanced datasets, without the need for extensive parameter tuning, renders it an appealing choice for financial institutions seeking reliable and efficient tools for credit risk assessment, financial analytics, and risk management.

The TSC-SVM model is a hybrid model. Therefore it is important to discuss the computational complexity of TSC-SVM model. First, the multi-view combined unsupervised method consists of three spectral clustering problems and a convex optimization problem. Spectral clustering’s computational complexity increases with sample points, but its overall complexity can be controlled. The Eq (3) which is a quadratic programming problem is computationally tractable. Second, semi-supervised SVM is a linear programming problem which is low computational costly. Despite the increase in computational complexity with sample size such as credit risk dataset, the overall computational complexity is manageable.

The Table 4 shows the computational time consumption in the 2018 Q3, 2018 Q4, Credit risk and Australian datasets. In summary, the computational complexity of the TSC-SVM model is affordable for most credit risk assessment datasets.

**Table 4:** Computational time consumption.

Dataset	Instances	First Stage	Second Stage	Overall
2018 Q4	13268	636s	2s	638s
2018 Q3	18819	705s	3s	708s
Credit risk	28638	990s	4s	994s
Australian	690	0.6s	0.1s	0.7s

### 4.3 Sensitivity analysis

**Fig 3:**
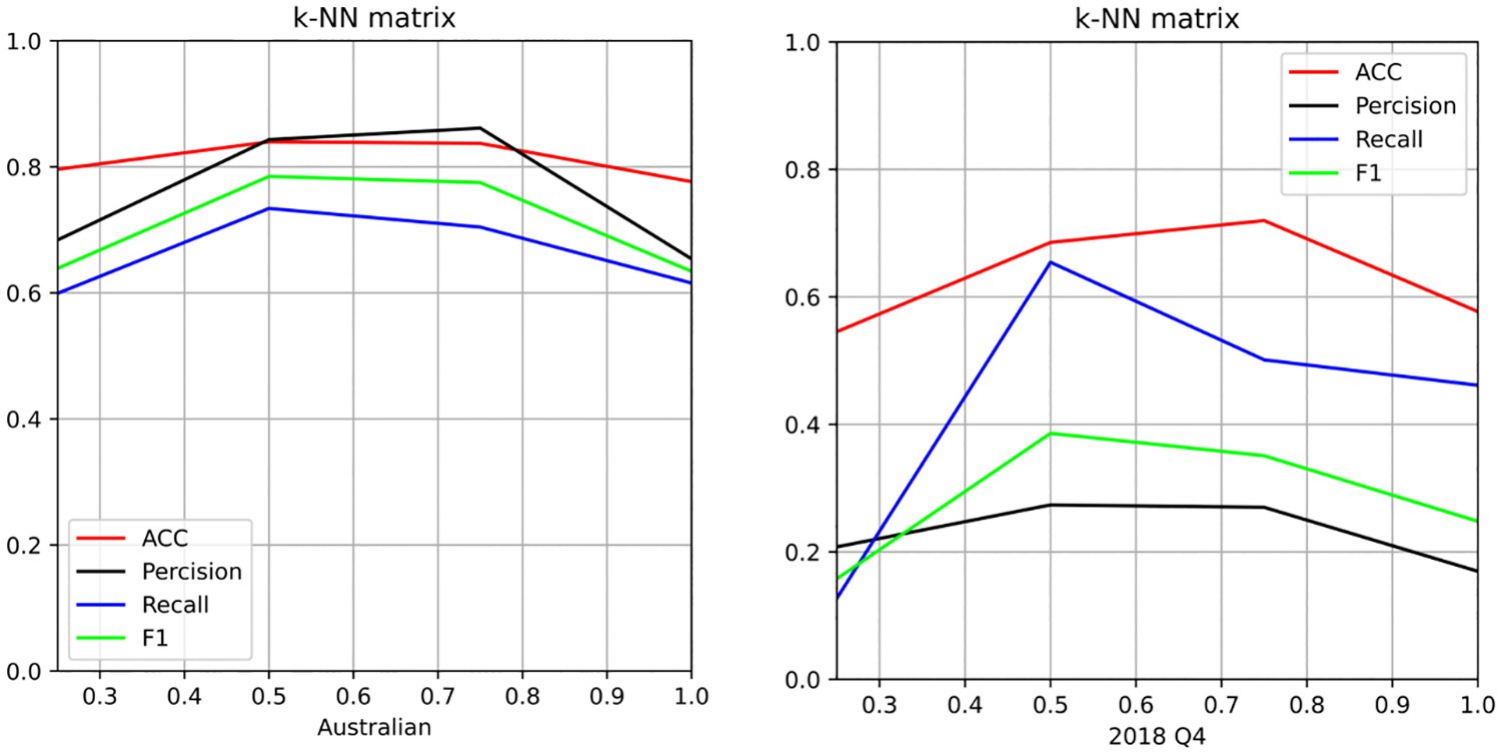
*k*-NN weighted adjacency matrix. Australian: it is the experiments results in Australian dataset with different parameter *k*. 2018 Q4: it is the experiments results in 2018 Q4 dataset with different parameter *k*.

To clarify the influence of the parameters on the model performance, additional experiments were conducted, focusing on the contributions of the *k*-NN weighted adjacency matrix, multi-view combined unsupervised method and penalty parameters. These experiments were designed to provide a fair evaluation of the model effectiveness across both balanced (Australian) and imbalanced (Lending Club 2018Q4) datasets. First, the influence of parameter *k* on the experimental outcomes was assessed, by varying *k* as a fraction of the total number of samples *n* in the dataset. The values of *k* were set as *k* = 0.25*n*, 0.5*n*, 0.75*n*, *n*. This range provided a comprehensive view of how the choice of *k* affects the model performance, especially in *k*-NN based methods, where the number of nearest neighbors (*k*) influences the model behavior. Fig 3 presents the results of these experiments, providing insights into the values of *k* that yield the optimal model performance in different types of datasets. These findings can guide users in selecting appropriate *k* values in practical applications. Such insights are particularly relevant for financial institutions and practitioners in credit risk assessment, aiding in fine-tuning the model to achieve the best possible balance between accuracy and robustness, tailored to the specific dataset characteristics.

Additionally, the experimental results provide valuable insights into the effectiveness of the *k*-NN weighted adjacency matrix compared with the fully connected weighted adjacency matrix in both balanced and imbalanced datasets: The *k*-NN weighted adjacency matrix consistently outperforms the fully connected weighted adjacency matrix across both types of datasets. This indicates that the *k*-NN matrix predicts more reliable and consistent outcomes, making it a preferable choice in practical applications. The *k*-NN matrix not only offers better performance but also requires less computational power compared with the fully connected weighted adjacency matrix. This observation aligns with reported results, highlighting the efficiency of the *k*-NN approach in handling large datasets [21]. Choosing an extremely small *k* value may adversely affect the model accuracy. A smaller *k* value may not capture enough information from the neighborhood, resulting in less reliable predictions. Imbalanced datasets exhibit greater sensitivity to the choice of *k* compared with balanced datasets. Thus, *k* must be carefully selected to ensure optimal performance. For binary classification problems, particularly in imbalanced contexts, *k* should ideally be more than half the total number of samples *n*. This settings can help ensure that the algorithm captures sufficient context from the data to make accurate predictions. These findings underscore the importance of carefully selecting *k* in *k*-NN based methods, especially in credit risk assessment, where the choice of parameters can significantly impact the model effectiveness. By adhering to these insights, practitioners can enhance the accuracy and reliability of their predictive models in various financial applications.

The influence of different perspectives in the multi-view combined unsupervised method must be examined to clarify their contributions to the model robustness and accuracy. The proposed method incorporates three distinct perspectives: forward view, backward view, and combined view, to thoroughly mine data for information, thereby enhancing the robustness and accuracy of label prediction. To explore the effectiveness of this multi-view approach, two additional experiments were conducted. First, labels were predicted using both the forward and backward views without combining them. This setup, referred to as the double view, enabled the assessment of how each perspective independently influences label prediction. Second, the forward and backward views were combined using the parameters (*μ*_1_, *μ*_2_) = (0.5, 0.5). This approach, termed average combining, was aimed at balancing the contributions of both views. Other parameters in the model were maintained at constant values to isolate the effect of the combination strategy. Comparing the results of these two experiments with the standard multi-view approach could provide insights into the advantages or potential limitations of integrating multiple perspectives. Specifically, this comparison could help understand whether combining the views leads to a significant improvement in performance compared with their independent use.

Table 5 presents the results of the experiments conducted using the double view and average combining strategies. Although the average combining algorithm achieves slightly better experimental results on the balanced dataset compared with the proposed method, the overall robustness of each approach must be considered. Similarly, the double view algorithm yields marginally better results on the imbalanced dataset. However, the robustness of the proposed method remains a significant advantage. The higher robustness of the proposed algorithm compared with both the double view and average combining algorithms is a critical factor, especially in real-world applications where datasets can vary significantly. This robustness is attributable to the ability of the proposed method in balancing the perspectives. The combining method based on perturbation theory ensures minimal discrepancy between the prediction results obtained from the three perspectives (forward, backward, and combined), enabling the achievement of consistent and balanced performance across different datasets and scenarios. These findings underscore the importance of a balanced approach that integrates multiple perspectives without allowing any one perspective to dominate the prediction process. Such a strategy is particularly beneficial in complex domains such as credit risk assessment, where the accuracy and reliability of predictions are paramount.

**Table 5:** Results of multi-view combined experiments.

		Accuracy	Precision	Recall	F1-Measure
2018 Q4	TSC-SVM	0.6854	0.2737	0.6544	0.3860
	Double view	0.7292	0.3031	0.6095	0.4048
	Average combining	0.6038	0.1880	0.4888	0.2716
Australian	TSC-SVM	0.8397	0.8432	0.7340	0.7848
	Double view	0.7765	0.6540	0.6156	0.6345
	Average combining	0.8412	0.8631	0.7143	0.7817

**Fig 4:**
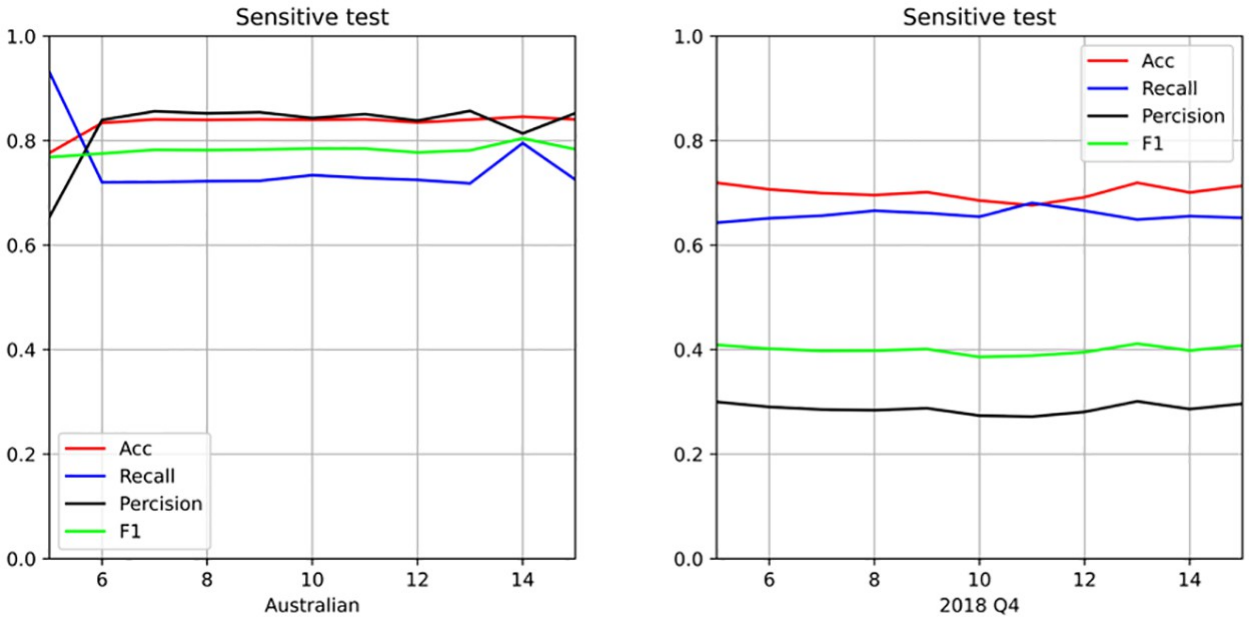
The sensitive test with penalty parameters. Australian: it is the experiments results in Australian dataset with different penalty parameter *c*_1_. 2018 Q4: it is the experiments results in 2018 Q4 dataset with different penalty parameter *c*_1_.

The purpose of the penalty parameters *C*_1_ and *C*_2_ is used to strengthen labeled sample constraints. Therefore, the robustness of the TSC-SVM model under these penalty parameters can be verified given the parameter *c*_2_. Since *C*_1_ must be sufficiently large, consider the value of *c*_1_ is given as *c*_1_ = 5, 6, …, 15. The Fig 4 shows TSV-SVM is robust to penalty parameters. Since it is designed to enforce the constraints of the support vector on the labeled samples. This means that in the unsupervised credit classification problem, financial institutions avoid the suffering of optimizing the penalty parameter. The necessary requirement is *C*_1_ ≫ *C*_2_. As a result, financial institutions are also able to reduce the cost of gathering labeled data and improve their profitability.

The TSC-SVM demonstrates strong robustness and accuracy when addressing unsupervised imbalanced credit risk assessment challenges. A key advantage of the TSC-SVM algorithm is its reduced sensitivity to parameters, which is valuable in practical applications where the optimal parameter settings may not be readily apparent or where data characteristics can vary significantly. The reduced need for extensive parameter tuning simplifies the model implementation, rendering it more user-friendly and accessible, especially for financial institutions with limited resources for such adjustments. By employing a robust and accurate model such as the TSC-SVM, financial institutions can expand their business scope. Specifically, the ability to more accurately assess credit risk in a variety of contexts and data environments can allow these institutions to explore new markets and customer segments with greater confidence. The enhanced performance of the TSC-SVM also translates into improved risk assessment capabilities. By providing more reliable and nuanced insights into potential credit risks, financial institutions can make better-informed decisions, ultimately leading to reduced losses owing to credit defaults and increased financial stability. Overall, the TSC-SVM offers a comprehensive solution for unsupervised imbalanced credit risk assessment, combining robustness, accuracy, and ease of use. These attributes make it an attractive option for financial institutions looking to enhance their analytical capabilities and risk management strategies.

## 5 Conclusions

This study introduces TSC-SVM, a novel approach designed for unsupervised imbalanced learning in credit risk assessment. The effectiveness and robustness of this algorithm are demonstrated through extensive testing on various credit datasets. The algorithm exhibits consistent performance across various credit datasets, indicating its robustness and adaptability to different data environments and scenarios. The key findings can be summarized as follows:

- Competitive performance in imbalanced credit datasets: The TSC-SVM model shows strong competitiveness with other well-established unsupervised classification techniques, including K-means, US-QSSVM, US-QSSVM-GOLD, PCut, Un-LDA, and spectral clustering. The proposed method can effectively identify minority samples in imbalanced datasets, which is particularly valuable in credit risk assessment, where accurately identifying potential defaulters is crucial for risk management and mitigating economic losses associated with loan defaults;
- Independence from prior knowledge for parameter adjustment: Unlike traditional models, the TSC-SVM algorithm does not rely on prior knowledge for parameter adjustment, making it easier for practitioners to implement and maintain without extensive tuning. Since the labeling of credit risk is time-consuming and labor-intensive. This can effectively help financial institutions classify the credit risk of unlabeled borrowers as quickly as possible;
- Global optimal solution achievement: The TSC-SVM model consistently finds the global optimal solution for all steps involved, effectively avoiding the pitfalls of local optimal. This aspect is vital for ensuring the overall accuracy and reliability of the model. This avoids financial institutions from suffering economic losses due to the model falling into local optimality;
- Enhanced robustness and accuracy through multi-view analysis: The multi-view combined unsupervised method, based on perturbation theory, significantly improves the model robustness and accuracy. By maintaining a balanced perspective among the forward, backward, and combined views, this method ensures that no single perspective disproportionately influences the overall prediction;
- Effective handling of unlabeled imbalanced data: The semi-supervised SVM component of the model employs strong constraints for labeled samples and weaker constraints for unlabeled samples. This approach effectively addresses the classification challenges posed by unlabeled imbalanced data, a common issue in credit risk datasets.

In summary, the TSC-SVM model presents a robust and effective solution for unsupervised imbalanced learning in credit risk assessment, offering financial institutions a powerful tool for enhancing their risk evaluation processes and reducing potential economic losses. In the future, we consider extending TSC-SVM algorithm to other fields which are suffering from the imbalanced problem such as disease diagnosis and fraud detection. In these fields, misclassifying minority samples costs more than misclassifying other samples. For example, improving the prediction accuracy of a few types of diseases or illnesses can effectively improve the treatment efficiency to patients.

## Supporting information

S1 Appendix Proof. The detailed mathematical derivations and the Karush–Kuhn–Tucker (KKT) conditions relevant to the semi-supervised SVM.
(PDF)
