# Supplementary material for: A hybrid unsupervised machine learning model with spectral clustering and semi-supervised support vector machine for credit risk assessment
Source: PLoS One. 2025 Jan 10;20(1):e0316557. doi: 10.1371/journal.pone.0316557 (PMC11723550; doi:10.1371/journal.pone.0316557)
Supplement: S1 Appendix — The detailed mathematical derivations and the Karush–Kuhn–Tucker (KKT) conditions relevant to the semi-supervised SVM. (PDF) [file pone.0316557.s001.pdf]

S1 Appendix. Proof

If the partial derivatives of L_{SVM} with respect to the primal variables (ω, b, ξ, σ) equal zero, the following expressions can be obtained:

$$\begin{cases} \omega = \sum_{i=1}^{n_1} \beta_i^1 y_i a_i + \sum_{i=1}^{n_2} (\beta_i^4 - \beta_i^3) q_i, \\ 0 = \sum_{i=1}^{n_1} \beta_i^1 y_i - \sum_{i=1}^{n_2} (\beta_i^4 - \beta_i^3), \\ C_1 = \beta_i^1 + \beta_i^2, \\ C_2 = \beta_i^3 + \beta_i^4 + \beta_i^5, \end{cases}$$

Parameter b can be computed using the KKT conditions, which state that the product between dual variables and constraints vanish at the optimal solution.

$$\begin{aligned} \min \quad & \frac{1}{2}||\omega||^2 + C_1 \sum_{i=1}^{n_1} \xi_i + C_2 \sum_{i=1}^{n_2} \sigma_i, \\ s.t. \quad & y(A\omega^T + b) \geq 1 - \xi, \\ & |Q\omega^T + b| \leq 1 + \sigma, \\ & \xi \geq 0, \quad \sigma \geq 0. \end{aligned} \tag{1}$$

For the optimal problem presented in Equation (1), the KKT conditions can be defined as follows:

$$\begin{cases} \beta_i^1 \geq 0, \quad ,\beta_i^2 \geq 0 \\ \beta_i^3 \geq 0, \quad \beta_i^4 \geq 0, \quad \beta_i^5 \geq 0, \\ y_i(a_i\omega^T + b) - 1 + \xi_i \geq 0, \\ q_i\omega^T + b + 1 + \sigma_i \geq 0, \\ -q_i\omega^T - b + 1 + \sigma_i \geq 0, \\ \beta_i^1(y_i(a_i\omega^T + b) - 1 + \xi_i) = 0, \quad \beta_i^2\xi_i = 0 \\ \beta_i^3(q_i\omega^T + b + 1 + \sigma_i) = 0, \\ \beta_i^4(-q_i\omega^T - b + 1 + \sigma_i) = 0, \quad \beta_i^4\sigma_i = 0 \\ \xi_i \geq 0, \quad \sigma_i \geq 0, \end{cases}$$
